# Tracking Solar Optimization in Renewable Energy Systems by Using Multiplexed Holograms in Bayfol^®^ Photopolymers

**DOI:** 10.3390/polym18060775

**Published:** 2026-03-23

**Authors:** Pedro Mas-Abellán, Pablo Beléndez, Jesús Gea-Caselles, José Carlos García-Vázquez, Belén Nieto-Rodríguez, Tomás Lloret, Inmaculada Pascual

**Affiliations:** 1 The Singular Theory (Singular Control Energy S.L.), Autovía de Alicante, km 52, 03400 Villena, Alicante, Spain; pmas@thesingulartheory.com (P.M.-A.); pablo.belendez@estudiantat.upc.edu (P.B.); jgea@thesingulartheory.com (J.G.-C.); 2 I.U. Física Aplicada a las Ciencias y las Tecnologías, Universidad de Alicante, Carretera San Vicente del Raspeig s/n, 03690 San Vicente del Raspeig, Alicante, Spain; jc.garciavazquez@ua.es (J.C.G.-V.); b.nieto@ua.es (B.N.-R.); pascual@ua.es (I.P.); 3 IES Mediterráneo, Avda. Tenerife 1, 03183 Torrevieja, Alicante, Spain; 4 Departamento de Física, Ingeniería de Sistemas y Teoría de la Señal, Universidad de Alicante, Carretera San Vicente del Raspeig s/n, 03690 San Vicente del Raspeig, Alicante, Spain; 5 Departamento de Matemática Aplicada, Universidad de Alicante, Carretera San Vicente del Raspeig s/n, 03690 San Vicente del Raspeig, Alicante, Spain; 6 Departamento de Óptica, Farmacología y Anatomía, Universidad de Alicante, Carretera San Vicente del Raspeig s/n, 03690 San Vicente del Raspeig, Alicante, Spain

**Keywords:** photopolymers, renewal energy, holography, multiplexed holograms, commercial photopolymer

## Abstract

Multidisciplinary technologies are truly driving major transformations in industries, innovating to become more efficient. The need for more efficient renewable energy systems, such as solar energy, has recently been addressed with the innovation of using holographic photonic devices to avoid solar tracking devices as much as possible. In this work, a multiplexed holographic device is created and characterized for use in front of a photocell, thereby eliminating the need for tracking systems due to its wide acceptance angle and high diffraction efficiency. Commercial Bayfol^®^ HX121 photopolymer was used as the holographic recording material to manufacture holograms, achieving high performance and facilitating the industrial scaling of this technique. Results obtained using the multiplexing technique enable low-frequency holograms (478 lines/mm) with a 43° acceptance angle. Using three of these devices, a 129° angular sweep is possible without the need for tracking.

## 1. Introduction

Throughout history, particularly in the last two centuries, energy has played a significant role in major economic changes. The three great industrial revolutions, responsible for transforming the world, have been based on the emergence of new energy sources.

The First Industrial Revolution, between the late 18th and mid-19th centuries, was marked by the beginning of the transition from traditional biofuels to the use of coal. The main cause of this change was the invention of the steam engine [1]. The Second Industrial Revolution, from the late 19th to the early 20th century [2], was marked by the emergence of oil and gas as new energy alternatives, which spurred the development and mass production of the automobile. Finally, the Third Industrial Revolution, beginning in the second half of the 20th century, was determined by the rise of digital and information technologies, communications, the automation of industrial processes, electronics, and renewable energies. It is undeniable that renewable energies have made significant progress, although they are still far from reaching their full potential and play a key role in the energy mix. The high existing demand for energy in the current world requires renewable energies to have the capacity to generate a positive impact in this context. However, these types of clean energy are based on relatively recent energy technologies, and therefore their use is not yet optimized [3,4,5]. Currently, the main energy objective is to achieve the goal of net-zero emissions [6]. To achieve this goal, technological development in the various renewable energy production systems is necessary in the coming years.

The Sun, as an inexhaustible source of energy, plays a key role in the transition to an environmentally friendly future. Its great potential, which remains largely unexplored, is undeniable and offers significant opportunities for the development of renewable energy solutions [7].

Each year, 4.4 × 10^16^ W from the sun falls on the Earth, a tremendous amount of power. Therefore, if solar energy could be converted into usable energy on a large scale, it would become the best potential solution to future energy needs.

In this regard, the question that must be addressed is whether all the energy the Sun provides is being used efficiently. To date, all applications requiring solar energy—since solar energy technology is still in its infancy—rely on placing the required device in the sun without any processing or transformation to maximize its performance. Due to this, inefficient results are obtained. It is worth noting that oil is not used directly after extraction but rather undergoes a refining process through which usable byproducts are extracted, such as fuels, tar, asphalt, lubricants, etc. [8]. This is because oil has been used as an energy source since the beginning of the 20th century, and the technology associated with its extraction and refining is highly mature.

The refining process allows for the creation of highly efficient energy systems. In this context, the following question arises: Why not do the same with sunlight? Why not create systems that optimize solar energy management, filtering it, selecting the necessary components, and redirecting it according to its intended use? [9,10].

Photovoltaic solar energy is an increasingly popular renewable energy source. One of the most important aspects of solar energy systems is the need to increase the efficiency of photovoltaic conversion by using devices that are not very costly.

In photovoltaic solar energy, the difference between theoretical and actual performance can be increased by various external factors: losses due to temperature [11], solar irradiance [12], the angle of incidence of solar radiation [13], dirt [14] or spectral losses [15], since photovoltaic cells exhibit spectral selectivity. To date, cooling systems to mitigate the negative effects of temperature on solar panel performance and tracking systems to follow the sun’s movement are commonly used. However, these measures maximize initial investment, maintenance, and operating costs, and do not provide a significant solution [16].

The use of holographic devices directly impacts the reduction in photovoltaic panel temperatures during the summer months, when very high temperatures are reached, and allows following the sun when several holographic elements are stored in the same place. Thus, the use of holographic elements could be a useful tool to manage the solar energy received by the photovoltaic panel [17].

Optics, and holography particularly, can contribute to improving the efficiency of photovoltaic systems at a lower cost [10,18,19,20,21,22]. Holographic solar devices use holographic optical elements as light-collecting systems. Among their advantages, it is worth noting that they are manufactured from thin-film materials, resulting in low weight. Furthermore, it is possible to record several of these elements on the same layer of material using multiplexing techniques [23]. In addition, they can be manufactured industrially using copying methods. Finally, once sealed, holographic optical elements (HOEs) can have a lifespan of between 25 and 30 years [10]. Consequently, the proposal advocates moving from the traditional “all or nothing,” “sun or shade” approach to the specific use of the sun’s different elements independently. This disruptive harnessing of solar radiation, which leads to highly efficient and productive processes, will be called solar refinement [24]. Solar refinement involves separating the different spectral components and modifying their properties through holographic engineering [10,25]. This would allow for the separate management of each band of solar radiation according to the needs of each situation. In the current context of the energy transition, greater utilization of solar radiation will allow for increased productivity and efficiency in clean energy generation through photovoltaics. For all these reasons, solar refinement is emerging as an indispensable tool for achieving the energy revolution sought by various countries.

Previous studies [25,26] characterized holographic lenses recorded onto photopolymers to concentrate incident light onto photovoltaic cells. When light does not reach the entire surface of the solar cell, it does not work properly. One way to cover the entire surface of the photocell is to use holographic gratings instead of holographic lenses.

In this paper, a multiplexed holographic grating was developed to direct spectral radiation onto the entire surface of a photocell, eliminating the need for solar tracking systems thanks to its wide acceptance angle and high diffraction efficiency. By increasing the acceptance angle, the operating range at maximum power is extended, or in other words, the system works at maximum power for a greater number of hours per day. Using Bayfol^®^ HX121 [27,28], a new commercially available photopolymer, high-performance multiplexed gratings were created due to the significantly greater thickness of the material compared to the commonly thinner Bayfol^®^ HX200 photopolymer [29]. Bayfol^®^ HX121 will enable the industrial scalability of this advanced technology.

## 2. Materials and Methods

The material used to manufacture multiplexed holographic concentrators is the commercial photopolymer Bayfol^®^ HX121 developed by Covestro AG (Leverkusen, Germany) [27]. This is a photosensitive, self-developing polymer film that allows the generation of volume and phase holograms [28]. Based on previous studies of photopolymers from the same manufacturer, such as Bayfol^®^ HX200 and Bayfol^®^ HX120 (Covestro, Leverkusen, Germany), the material is known to exhibit good thermal stability, with degradation processes occurring at temperatures well above the typical operating conditions of holographic optical elements [28,29,30].

Suitable materials for holographic recording must exhibit high energy sensitivity to the laser wavelengths used, high resolution, linear recording behavior, low noise, and an economical price. Since the beginnings of holography, various recording materials have been used, such as photographic emulsions, dichromated gelatins, photoresist, photorefractive, photochromic, and photothermoplastic materials [31]. However, thanks to their great versatility, photopolymers are the most widely used holographic recording materials today [32].

Photopolymers are organic substances (monomers) that polymerize under the influence of light, forming long polymer chains. The interference fringes that form in the material originate directly from a change in the refractive index as it transitions from monomer to polymer. Furthermore, these materials do not require chemical processing, so the holograms are formed in real time. Another advantage of photopolymers compared to other holographic recording materials is their ease of handling after exposure, as they only need to be cured with a suitable light source to be fixed. Additionally, depending on the dyes included in their composition, they can be made sensitive to the entire visible spectrum [33,34,35,36]. One of the photopolymers traditionally used in holography has been polyinyl alcohol/acrylamide materials, whose behavior has been studied both experimentally and theoretically [37,38,39,40]. These photopolymers have been also used in numerous holographic applications [41,42]. Holographic photopolymers with a sustainable design have also been developed [33,36,43,44]. However, the Bayfol^®^ photopolymers from the Covestro company are currently receiving the most attention in the literature [27,28].

Bayfol^®^ HX121 can be etched with monochromatic, coherent laser light within the visible spectral wavelength range of 440 nm to 680 nm. It is 30 µm thick and has a refractive index of 1.50. The highest transmission for Bayfol^®^ HX121 occurs in the blue region and the lowest in the red zone around 650 nm [27]. In the green zone (specifically at 532 nm), HX121 exhibits low transmission, resulting in high absorption. This means that little power is required to create holograms when using this wavelength for recording holograms, which is the wavelength used in this work.

**Figure 1:**
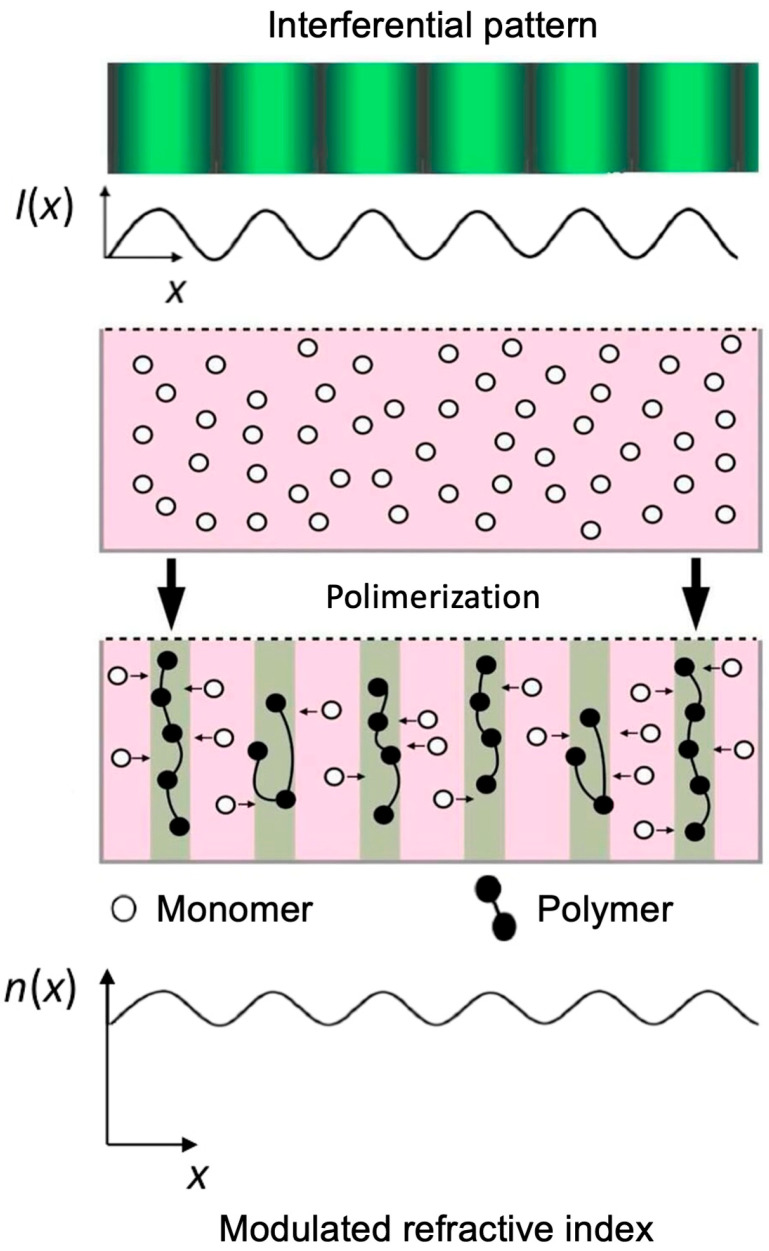
Polymerization process and refractive index modulation. Green strips are the polymerized areas, which correspond to the illuminated zones, and pink strips are the dark areas of the stored interference pattern.

These materials absorb light when exposed to a specific wavelength, which excites the dye and activates the initiator. The initiator generates free radicals that react with the monomer, producing the polymerization reaction. The polymer chains generated in the polymerization reaction are responsible for the changes in the properties of these materials. The hologram is stored by the variation of these properties [45]. The hologram formation mechanism occurs due to the modulation of the refractive index between the polymerized and non-polymerized areas, which correspond to the illuminated and dark areas, respectively, of the stored interference pattern (Figure 1).

To provide mechanical stability, the recording material adheres to a glass support on the photopolymer face without the need for adhesive. This is a requirement when high frequencies in the interference pattern are recorded.

The experimental device for holographic recording is placed on a holographic table equipped with an anti-vibration system that isolates it from the floor, thereby preventing any vibration from being transmitted to the material and potentially damaging the recording.

**Figure 2:**
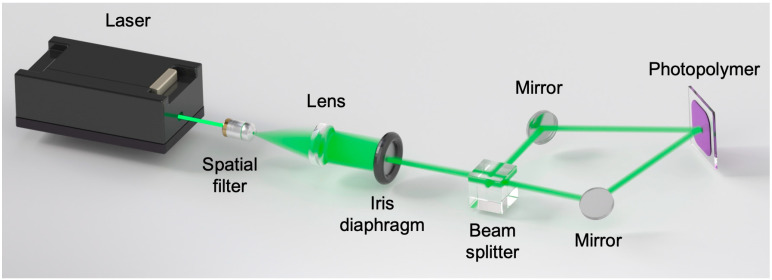
Schematic of the experimental device used in the recording stage.

The experimental setup used in the holographic grating recording stage for solar refining is shown in Figure 2. The experimental setup consists of a Cobolt diode continuous-wave laser source with three independently selectable emission wavelengths: 473 nm, 532 nm, and 660 nm. The nominal power ratings are 300 mW, 500 mW, and 500 mW, respectively. For holographic recording, the beam is “cleaned” using a spatial filter, and then it is collimated with a lens. After that, it is split into two beams (object and reference beam) using a 1:1 beam splitter. Both reference and object beams are directed onto the material after being reflected by different mirrors, resulting in an interference pattern on the photopolymer.

The size of the spot and subsequent hologram is 1 cm in diameter. The object and reference recording angles (*θ_o_* and *θ_r_*) have been measured with respect to the normal to the recording material, with *θ_r_* = −*θ_o_* = 7.30° for low frequency holographic gratings (478 lines/mm) and *θ_r_* = −*θ_o_* = 18.70° for high frequency gratings (1205 lines/mm).

It is worth noting that, after the registration stage, a curing process is carried out using white light from a LED lamp (6500 K, 875 lm) placed 37.5 cm from the sample for five minutes. This is performed to stop the polymerization process and bleach the remaining dye from the sample The lamp is located inside a sealed chamber so that the radiation reaches only the sample.

Hologram multiplexing [46] consists of storing several holograms at the same position on the material by varying some parameter without the holograms overlapping. Depending on the parameter varied and how these holograms are stored, multiplexing methods can be classified as follows:

- Angular multiplexing: the wavelength is changed for each recording.
- Peristrophic multiplexing: the recording material is rotated about an axis perpendicular or parallel to the recording plane.
- Phase-coded multiplexing: the phase of the reference wave is modified.

**Figure 3:**
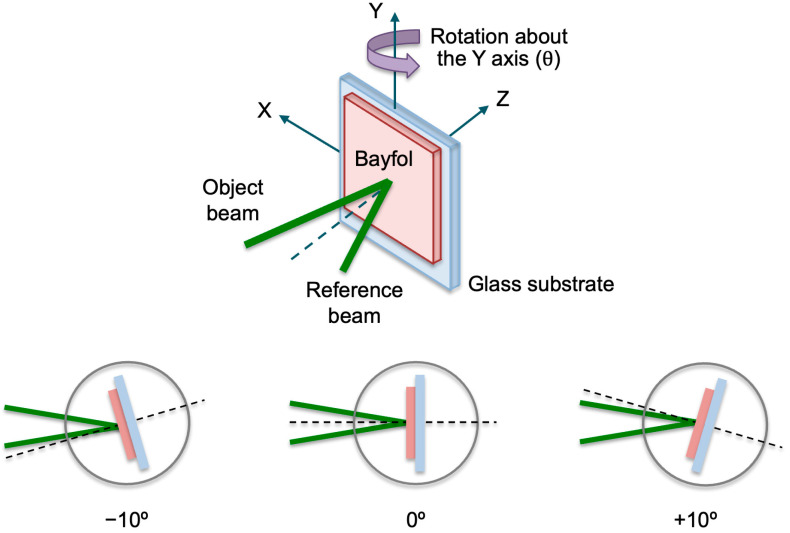
Diagram of the movement of a hologram to perform peristrophic multiplexing and geometric diagram of the records of three peristrophically multiplexed diffraction gratings.

In this work, diffraction gratings were multiplexed peristrophically, where the multiplexing was performed by rotating the material about an axis of rotation parallel to the material (*Y*-axis) and, therefore, perpendicular to the recording plane. Figure 3 shows a schematic of peristrophic multiplexing and the different plate positions for multiplexing three holograms [47]. In this paper, the plate has been rotated by −10°, 0°, and +10°, and by −8°, 0°, and +8°.

**Figure 4:**
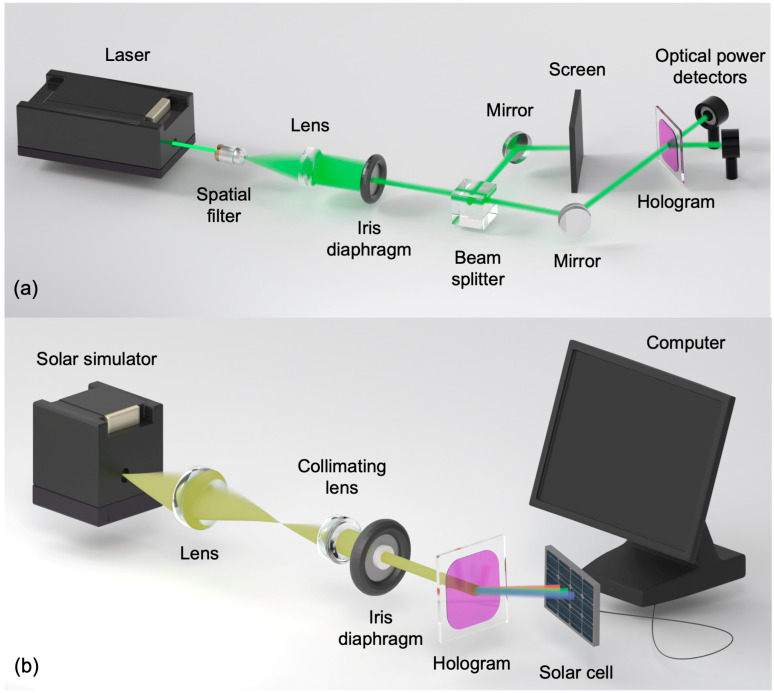
(**a**) Experimental device used in the reconstruction (**a**) with the laser (532 nm) and (**b**) with the solar simulator AM1.5G.

Two different experimental setups were used to reconstruct the holographic diffraction gratings. First, the HOEs were reconstructed using the same laser used for recording, as shown in Figure 4a and measured the power of the diffracted beam by using an optical power meter (Newport Model 2935-C, Darmstadt, Germany) and an optical power detector (Newport Model 918D-SL-OD3R, Darmstadt, Germany). Second, the experimental setup used to reconstruct the HOEs with the solar simulator is shown in Figure 4b. The fabricated HOEs were characterized using a broadband, non-polarized source and a monocrystalline silicon photovoltaic cell, measuring the relative current under short-circuit conditions (*R* = 0 Ω). The solar simulator source emits a continuous AM1.5G solar spectrum (350–1800 nm) and operates with a collimated beam. The beam diffracted by the HOE hits the photovoltaic cell as a spectrum-split beam. This is because each wavelength is diffracted at a different diffraction angle.

## 3. Experimental Results and Discussion

**Figure 5:**
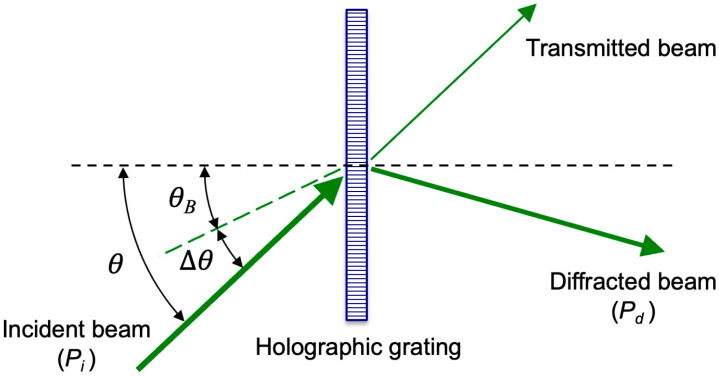
Parameters used in the reconstruction of holographic gratings.

A holographic concentrator consists of at least one HOE capable of directing diffracted light, which constitutes the extended spectrum, onto the solar cell area [10]. Diffraction efficiency (*η*) describes the amount of optical power diffracted by a hologram in the direction of the diffracted beam. It is defined as the power of the diffracted beam relative to the power incident on the hologram [48]:

(1) $$\eta \left(\%\right)=\frac{P_{d}}{P_{i}}\;\times 100$$

where *P_d_* is the optical power diffracted in the direction of diffraction and *P_i_* is the total power incident on the hologram (Figure 5). If *θ_B_* is the Bragg angle, the angular deviation Δ*θ* with respect to the Bragg angle is defined by:

(2) $$\Delta \theta =\theta -\theta_{B}$$

where *θ* is the angle formed by the reconstruction beam with respect to the normal to the hologram, so when *θ* coincides with the Bragg angle *θ_B_* we have Δ*θ* = 0°.

A laser with a wavelength of λ_r_ = 532 nm was used at this stage. The holographic gratings were recorded at different exposure times, which, multiplied by the laser recording intensity (5 mW/cm^2^), gave different exposures. The grating characteristics are shown in Table 1.

**Figure 6:**
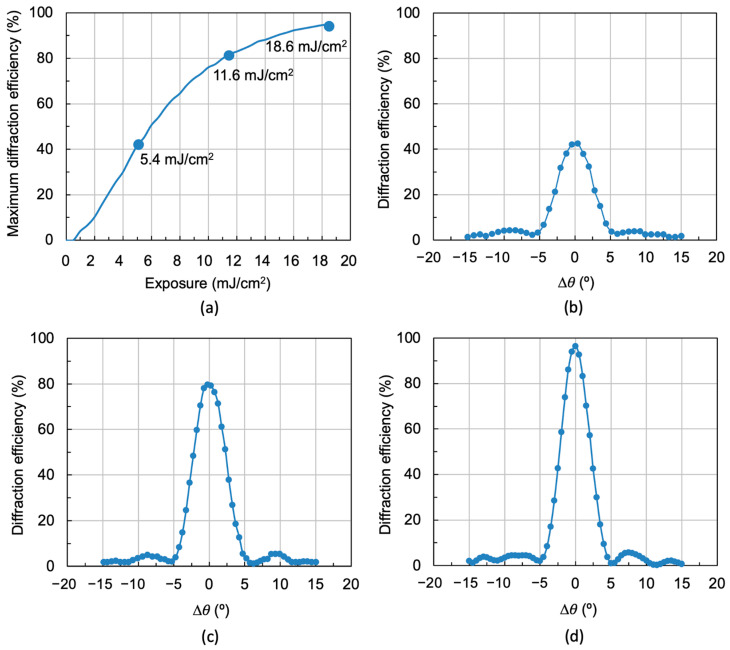
(**a**) Maximum diffraction efficiency versus exposure. The grating characteristics are shown in Table 1. The solid line is a guide for the eye. Diffraction efficiency versus angular deviation for the exposures: (**b**) 5.4 mJ/cm^2^, (**c**) 11.6 mJ/cm^2^ and (**d**) 18.6 mJ/cm^2^.

Figure 6a shows the maximum diffraction efficiency versus exposure. Three exposure values are indicated in this figure: 5.4 mJ/cm^2^, 11.6 mJ/cm^2^ and 18.6 mJ/cm^2^. These are symmetrical holographic gratings (*θ_o_* = −*θ_r_*) stored on Bayfol^®^ HX121 recording material, and their characteristics are shown in Table 1. Figure 6b–d shows the diffraction efficiency versus angular deviation Δ*θ* for the holographic gratings of the three exposures shown in Figure 6a. The reconstruction was carried out with the 532 nm wavelength laser according to the scheme in Figure 4a.

Figure 6d shows a maximum diffraction efficiency of 97%, which means that the light incident on the hologram is diffracted and almost no light is transmitted. However, Figure 6b,c shows holograms with lower diffraction efficiency, so some of the light is transmitted and some is diffracted.

When the solar simulator is used to reconstruct the holographic diffraction grating, white light is incident on it at an angle of incidence *θ_i_*. The diffraction grating decomposes the white light and the different component colors of the solar radiation spectrum appear angularly distributed (extended spectrum) in the diffracted beam. This is because each wavelength *λ* is diffracted with a different diffraction angle *θ_d_*. In the most general case, the diffraction angle *θ_d_* is [49]:

(3) $$\sin\theta_{d}=\sin\theta_{i}-\frac{\lambda }{d_{x}}$$

where *d_x_* is the distance between the Bragg planes measured along the *X*-axis. For symmetric holographic gratings, *d_x_* = Λ is fulfilled and the diffraction angle *θ_d_* is calculated by:

(4) $$\sin\theta_{d}=\sin\theta_{i}-\frac{\lambda }{\Lambda }$$

**Figure 7:**
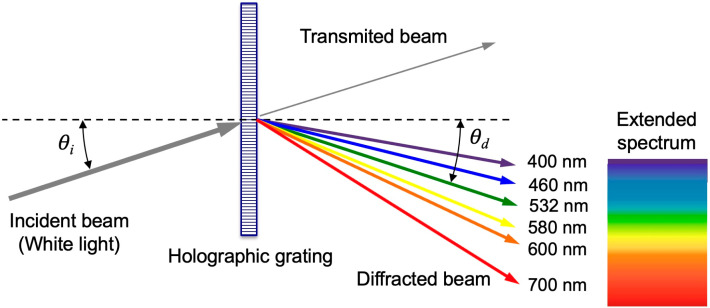
Reconstruction of a holographic diffraction grating with white light. Each wavelength is diffracted with a different diffraction angle *θ_d_*.

For more information on the geometric characterization of holographic transmission gratings, see Appendix A. Figure 7 shows a schematic of the reconstruction of a symmetrical holographic grating when reconstructed in white light.

**Figure 8:**
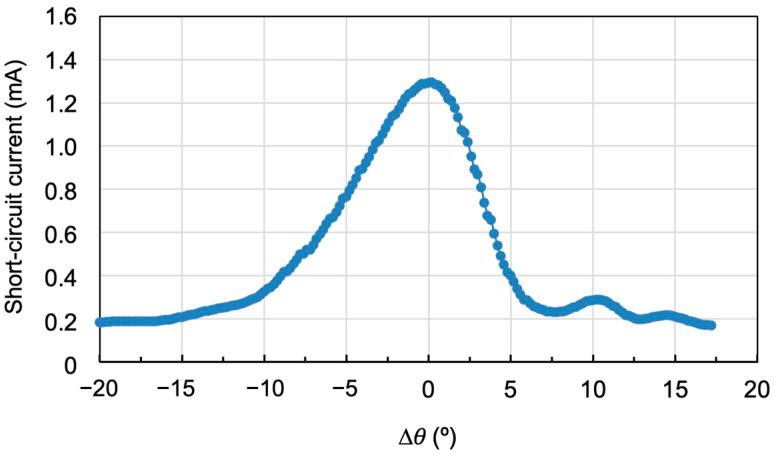
Reconstruction with the solar simulator of the holographic grating in Figure 6d.

Figure 8 shows the reconstruction of the hologram in Figure 6d using the solar simulator (see diagram in Figure 4b). As it was seen in Figure 6d, when the reconstruction is carried out with the laser, a maximum diffraction efficiency of 97% is achieved, but the angular range of the diffraction grating is limited to 10°. However, when illuminated with the solar simulator (Figure 8), the angular range is increased, reaching a value of the order of 18°. This angular width is known as the acceptance angle and corresponds to the angle of the solar rays that can strike the hologram and be diffracted towards the solar cell. It is concluded that when the hologram is illuminated with the solar simulator, the acceptance angle is higher than when it is illuminated with the laser (532 nm). This is because this angle depends on the reconstruction wavelength. In other words, under real operating conditions, the area under the curve would represent the effective energy contribution of the system over the course of the day in a photovoltaic system.

From Coupled Wave Theory [50], it can be deduced that a low film thickness and a low spatial frequency are key parameters for achieving a wide acceptance angle using a single hologram. The recording material used is Bayfol^®^ HX121, with a thickness of *d* = 30 µm. In addition to the previously discussed 478 lines/mm spatial frequency holographic diffraction gratings, 1205 lines/mm spatial frequency holographic gratings have also been recorded (Table 2).

A high spatial frequency (*f* = 1205 lines/mm) ensures that the hologram is volume hologram (*Q* = 97 > 10) [48] and then all the diffracted light ends up in a single diffraction order. By decreasing the spatial frequency (*f* = 478 lines/mm) and working with small thicknesses, *Q* = 15 is obtained (Table 1) and the holograms approach the limit marked by the *Q* factor > 10, but they are still volume holograms. The strategy of reducing the thickness of the recording material is limited by the thickness of the commercial material, which is fixed, and the reduction in the spatial frequency is limited by the *Q* factor. For this reason, a multiplexed holographic system has been developed in which more than one diffraction grating has been recorded at the same location on the material. The peristrophic multiplexing technique will be used so that the diffracted sunlight reaches the photocell from different angles of incidence, since the diffraction gratings will be recorded at different angles. In this way, the effect of each of the diffraction gratings considered is combined, and the acceptance angle is increased.

**Figure 9:**
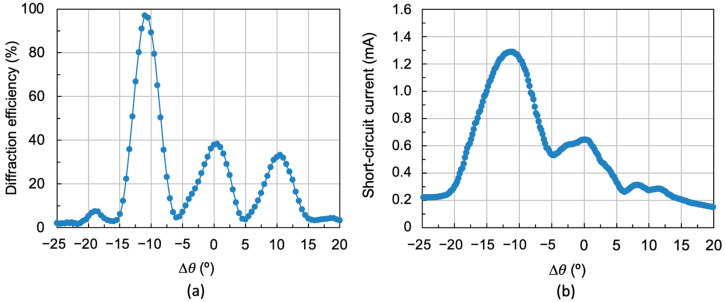
(**a**) Diffraction performance as a function of angular deviation when using the laser to reconstruct the three multiplexed holographic gratings separated by 10° between peaks and with exposures of 20 mJ/cm^2^ for each grating, and (**b**) short-circuit current as a function of angular deviation when reconstructed with the solar simulator for the three multiplexed holographic gratings separated by 10° between peaks.

To determine the separation between two consecutive diffraction gratings in peristrophic multiplexing, it is necessary to consider the angular width of a single grating. For the grating with spatial frequency *f* = 478 lines/mm in Figure 6d, the angular width is 10°. This will be the angular separation between each two consecutive diffraction gratings so that their diffraction maxima are sufficiently separated. In this way, the diffraction gratings will act independently. Three holographic diffraction gratings with a constant angular separation of 10° were stored following the recording scheme in Figure 3. Exposure times were 4 s for each grating, corresponding to an exposure of 20 mJ/cm^2^. Next, once the gratings were cured using white light from a LED lamp, the multiplexed gratings were reconstructed with both the 532 nm wavelength laser and the solar simulator. The results obtained are shown in Figure 9.

Figure 9a shows an angular response of 30° for the three multiplexed gratings reconstructed with the 532 nm laser. However, the diffraction efficiency of the gratings is highest for the first grating, 97%, and only around 40% for the last two. This is because multiplexing requires adjusting the exposure times: shorter for the first gratings and longer for the last. This is necessary for all diffraction gratings to achieve high efficiency. Otherwise, recording the first grating would consume all the dye, making it impossible to record the remaining gratings with high efficiency.

Figure 9b shows the angular response of the same multiplexed gratings when reconstructed using the solar simulator. The acceptance angle is 35°, but the short-circuit current decreases, especially for positive angles, which correspond to the gratings with the lowest diffraction efficiency. Therefore, it is important to find a compromise between an adequate acceptance angle and good diffraction efficiency, resulting in a high short-circuit current when diffracting light toward the solar cell. This will allow for the fabrication of optimal holographic solar concentrators.

**Figure 10:**
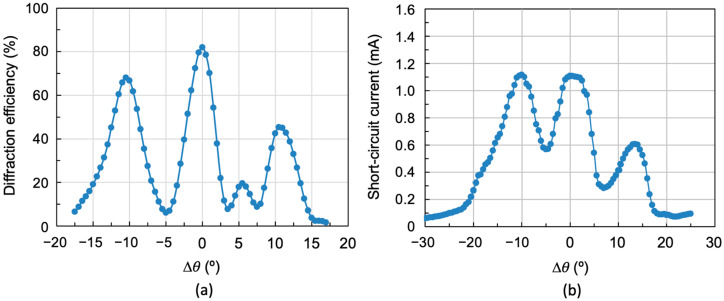
(**a**) Laser reconstruction of three multiplexed gratings separated by 10° between peaks and with exposure times set to 3, 6 and 20 s, exposures of 15, 30, and 100 mJ/cm^2^, respectively, and (**b**) reconstruction with the solar simulator of the same three multiplexed gratings.

Figure 10 shows the results obtained when three multiplexed gratings separated by 10° are reconstructed again, but now the exposure times have been set to 3, 6, and 20 s, respectively (exposures of 15, 30, and 100 mJ/cm^2^). Figure 10a shows the diffraction efficiency as a function of angular deviation Δ*θ* using a laser light source (532 nm), with an acceptance angle of 33° and maximum diffraction efficiency of 70%, 82%, and 49%, respectively, with a mean of 67%.

Figure 10b shows the same angular response using the solar simulator in the reconstruction. As can be seen, there is an increase in the acceptance angle, which is now 43°. Three short-circuit current intensity peaks corresponding to the three multiplexed gratings can be distinguished. Although there is a decrease in short-circuit current between peaks, it does not reach zero. Comparing Figure 10a and Figure 10b, it can be concluded that when reconstructing with a monochromatic laser, the diffraction efficiency drops are pronounced and almost zero. However, when reconstructing with the solar simulator, other wavelengths appear to impinge on the photocell, generating a certain short-circuit current, which means the intensity drops do not reach zero.

**Figure 11:**
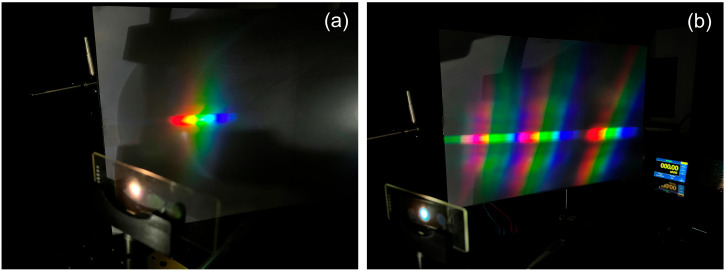
Extended spectrum obtained by reconstructing the holographic grating with the solar simulator (white light): (**a**) individual from Figure 6d, and (**b**) multiplexed from Figure 10b.

Figure 11 shows the spectrum generated by both the individual holographic grating and the three multiplexed gratings.

**Figure 12:**
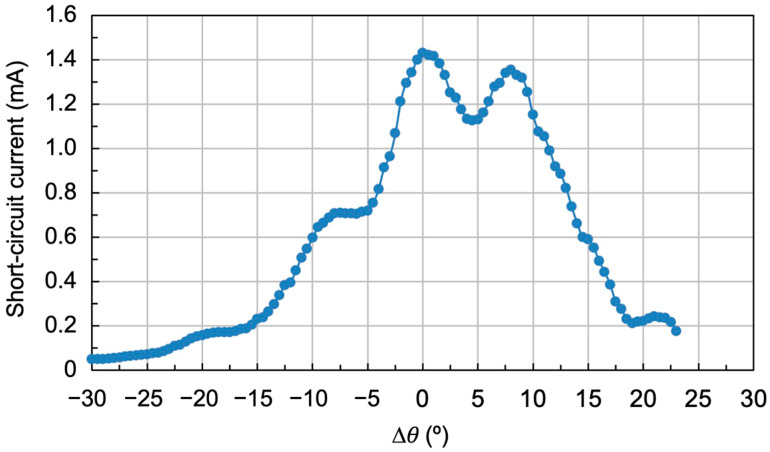
Reconstruction using the solar simulator of three multiplexed gratings with exposures of 15, 30, and 100 mJ/cm^2^, separated by 8° between peaks.

To try to reduce these intensity dips between peaks, three multiplexed holograms were again recorded with the same exposures of 15, 30, and 100 mJ/cm^2^, and with an angular separation between peaks of 8°. The results are shown in Figure 12. As can be seen, there is virtually no dip between peaks, although the acceptance angle is somewhat smaller, in this case between 35° and 40°.

The number of diffraction gratings that can be multiplexed is determined by the thickness of the recording material. If more holographic diffraction gratings are to be multiplexed, the thickness of the material must be increased. Since we are working with a commercial material, the thickness cannot be modified as it is fixed by the manufacturer. Therefore, it was decided to vary the spatial frequency by increasing it. Although increasing the spatial frequency from 478 lines/mm to 1205 lines/mm in a symmetrical hologram decreases the width of its angular response [46], it also results in a decrease in the dip between peaks.

**Figure 13:**
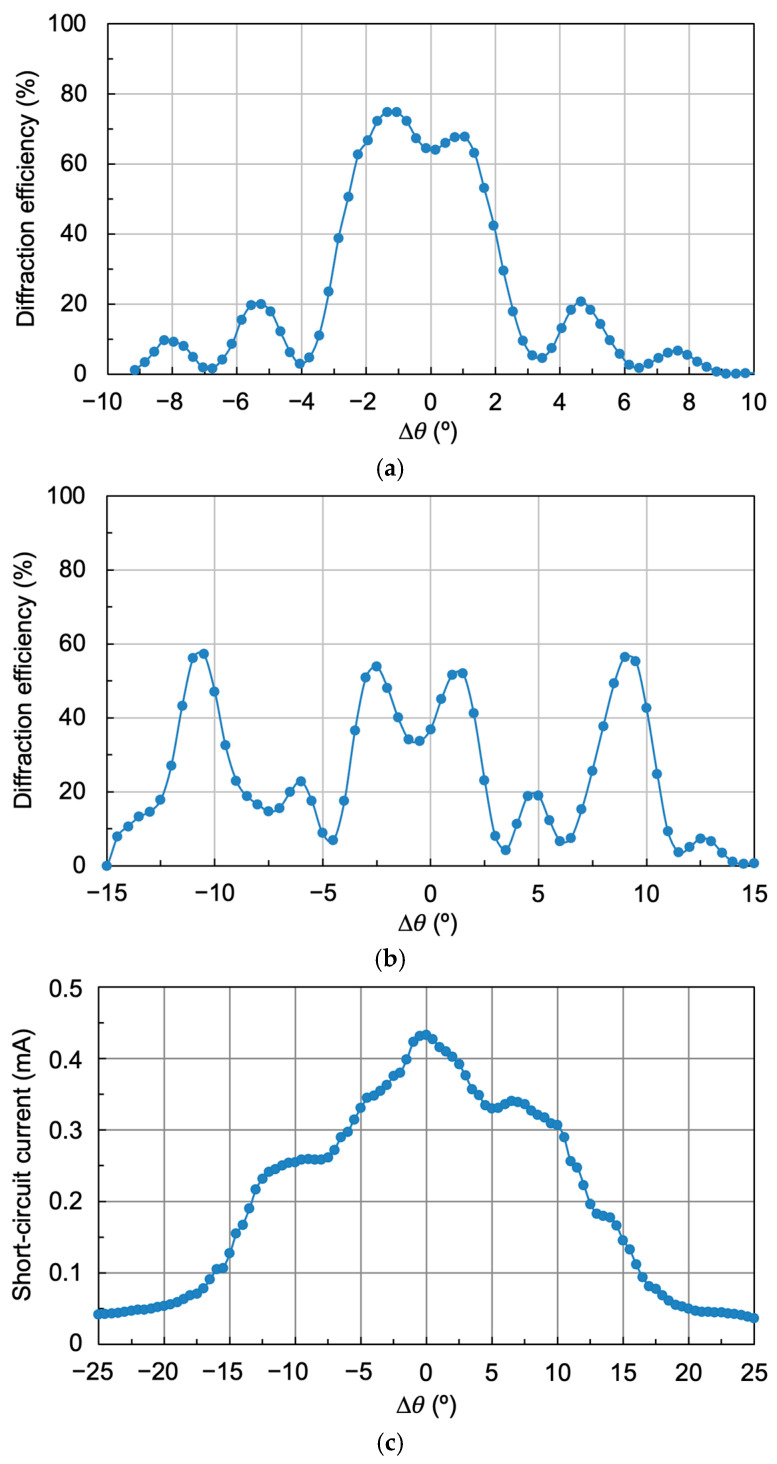
(**a**) Angular response of 1205 lines/mm frequency holographic gratings, reconstructed with the laser, (**b**) laser reconstruction of three multiplexed gratings separated by 10° between peaks and with exposure times set to 3, 6, and 20 s, exposures of 15, 30, and 100 mJ/cm^2^, respectively, and (**c**) reconstruction using the solar simulator of the three multiplexed gratings.

Diffraction gratings were recorded with a frequency of 1205 lines/mm, and their angular response is shown in Figure 13a. As can be seen, the maximum diffraction efficiency achieved is 75%, but its angular width is somewhat smaller, around 7.5°, as expected. Two peaks appear in the region of maximum efficiency due to the curing process.

Three diffraction gratings were then multiplexed in the same area of the recording material, using the same exposure times: 3, 6, and 20 s, and exposures of 15, 30, and 100 mJ/cm^2^, respectively. The separation angles between gratings were 10°, the same as those used to record the gratings in Figure 10a. Figure 13b shows the diffraction efficiency versus angular deviation. The first thing to notice is that all the stored gratings have the same diffraction efficiency, around 55%. Furthermore, the central grating presents two peaks at the top due to the curing process.

Figure 13c shows the reconstruction results of the multiplexed gratings with the solar simulator (short-circuit current versus reconstruction angle). As can be seen, there are no drops in intensity between peaks, with the acceptance angle being approximately 40°. However, the short-circuit current is somewhat lower than that obtained for the lower frequency due to the angular separation between diffraction orders.

**Figure 14:**
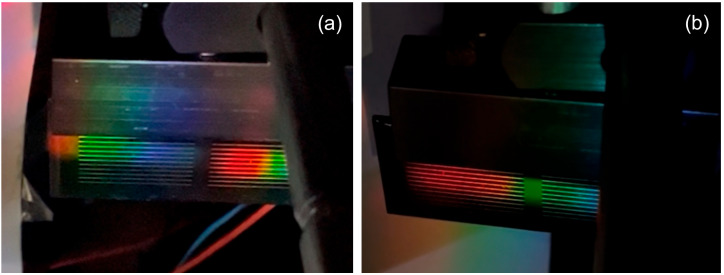
Extended focus impinging on the photocell: (**a**) low frequency multiplexed hologram, two of the first diffraction multiplexed orders, and (**b**) high frequency multiplexed hologram, only one of the first diffraction multiplexed orders.

At low frequency, the first diffraction multiplexed orders reach the photocell simultaneously (at least two of them), as in Figure 14a; however, at high frequency, as in Figure 14b, the first diffraction multiplexed orders do not reach the photocell simultaneously due to the angular separation between multiplexed orders (only one of them).

In summary, for low spatial frequency (478 lines/mm), the best results in terms of generated short-circuit current (1.1 mA) and acceptance angle (43°) are obtained for three multiplexed gratings separated angularly by 10° between peaks and with exposures of 15, 30, and 100 mJ/cm^2^ (Figure 10b). In the case of high spatial frequency (1205 lines/mm), the best results in terms of uniformity of the generated short-circuit current (0.44 mA) and acceptance angle (40°) are obtained for three multiplexed gratings separated by 10° between peaks and with exposures of 15, 30, and 100 mJ/cm^2^ (Figure 13b).

Finally, to evaluate the practical performance of the system formed by a photocell with a multiplexed hologram, it is important to consider that the performance of a photocell under real operating conditions is affected by several environmental factors, including temperature variations, changes in light incidence, and seasonal effects. The implementation of a multiplexed holographic grating system can mitigate some of these losses in the photocell. In particular, the use of multiplexed holographic gratings helps maintain near-normal light incidence on the photocell for longer periods of time, allowing it to work at maximum power for more hours a day. As a result, incidence-related losses are reduced, leading to an overall improvement in photocell performance. Thus, a cost analysis was conducted to estimate the manufacturing costs of these devices. The unit cost depends on the production scale, taking into account economies of scale; under typical assumptions, the estimated price could range from approximately EUR 10 to EUR 50/m^2^, or potentially lower. Additionally, the system does not require conventional solar tracking mechanisms. An important advantage of this approach is that the multiplexed holographic structure effectively performs the tracking function itself, enabling solar tracking without moving parts and therefore without associated maintenance costs.

## 4. Conclusions

In conclusion, this article presents the characterization of the commercial photopolymer Bayfol^®^ HX121 as a master material for holographic optical elements intended for photovoltaic applications. To the best of our knowledge, this is the first study to describe its characterization for this type of application. The relatively large thickness of the material (~30 μm), compared to other photopolymers from the same manufacturer, Covestro, such as Bayfol^®^ HX200, makes it particularly suitable for multiplexing techniques, as it allows for the recording of multiple holographic diffraction gratings. Furthermore, unlike previous approaches based on multiplexed holographic lenses, this work explores the multiplexing of diffraction gratings in order to distribute incident solar radiation over a larger surface area of the photovoltaic cell. The results demonstrate that this approach promotes more uniform illumination of the cell surface, allowing the device to operate under more favorable conditions.

On the other hand, holographic technology is presented as an exceptional method for obtaining efficient, thin, economical, and versatile diffraction gratings capable of operating at a wide acceptance angle. The possibility of directing sunlight toward the solar cell with a wide acceptance angle and without the need for mechanical movement (solar tracking system) has been demonstrated. A system comprising a photocell and a three-multiplexed holographic grating (478 lines/mm spatial frequency), separated angularly by 10° between peaks, can deflect sunlight onto the photocell with an acceptance angle of 43°, maintaining short-circuit current variations without dropping to zero within this range. Using three of these devices, a 129° angular sweep is possible without the need for tracking.

The findings of this paper highlight the potential of this photopolymer for the development of multiplexed holographic gratings in photovoltaic applications, while its commercial availability also makes it attractive for future industrial implementation. In future works, a more in-depth study will be conducted on how to improve the practical performance of the photocell-hologram system when it is affected by environmental factors, or by seasonal effects.

## Figures and Tables

**Table 1:** Parameters of holographic gratings with spatial frequency 478 lines/mm.

Reference beam angle (*θ_r_*)	7.30°
Object beam angle (*θ_o_*)	−7.30°
Spatial frequency (*f*)	478 lines/mm
Grating period (Λ)	2.09 µm
*Q*-factor [45]	15

**Table 2:** Parameters of holographic gratings with spatial frequency 1205 lines/mm.

Reference beam angle (*θ_r_*)	18.70°
Objetc beam angle (*θ_o_*)	−18.70°
Spatial frequency (*f*)	1205 lines/mm
Grating period (Λ)	0.83 µm
*Q*-factor [45]	97

**Figure A1:**
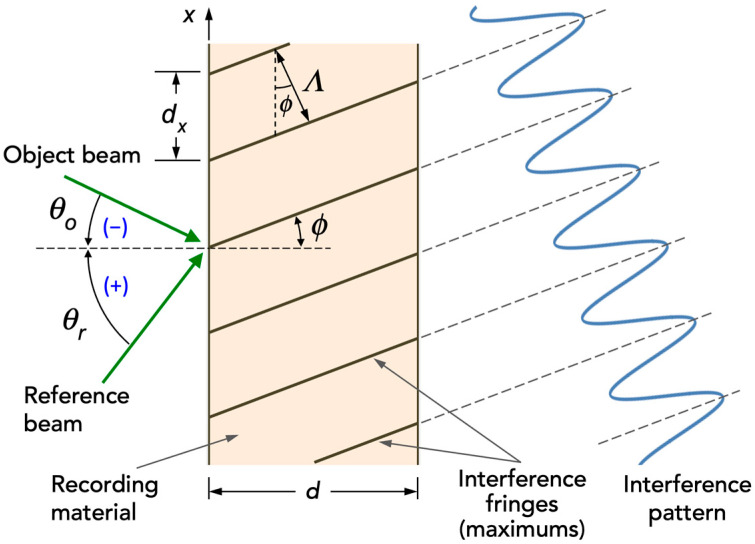
Definition of the parameters used to characterize a holographic transmission diffraction grating.

**Figure A2:**
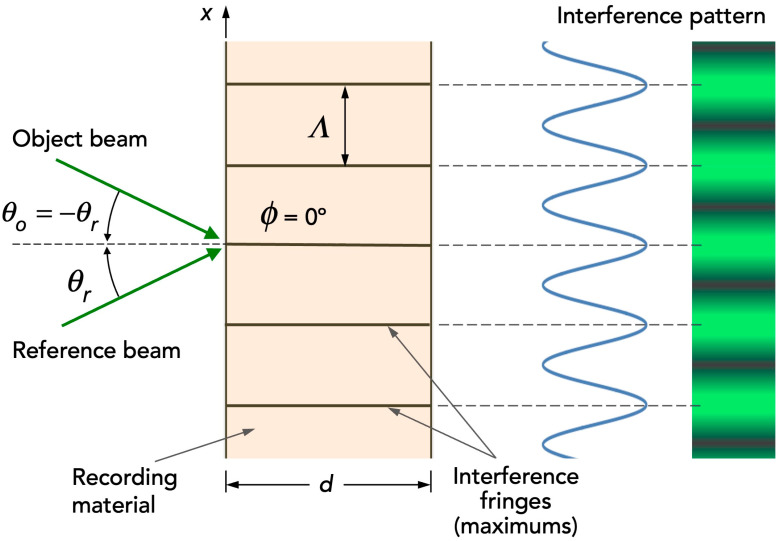
Definition of the parameters used to characterize a symmetric holographic transmission diffraction grating.

## Data Availability

The original contributions presented in this study are included in the article. Further inquiries can be directed to the corresponding author.

## Appendix A. Holographic Transmission Diffraction Gratings

In this paper, holographic transmission diffraction gratings have been considered, which behave as both volume and phase holograms. In a transmission holographic grating, the object and reference beams strike the same face of the material and, therefore, travel in the same direction. If a hologram is a volume hologram [48], upon reconstruction only two beams emerge: the diffracted beam and the transmitted beam, the latter in the direction of the reconstruction beam. Finally, a phase hologram modifies the phase of the reconstruction wave, but not its amplitude. In the case of the phase holograms analyzed in this paper, this has been achieved through periodic modulation of the refractive index of the material when illuminated by the light interference pattern.

When two plane beams (object beam and reference beam) of wavelength *λ_r_* are incident on a holographic recording material of refractive index *n* and thickness *d* (Figure A1), their interference pattern gives rise to a set of interference fringes whose maxima are parallel and equidistant planes known as Bragg planes [48]. The separation *d_x_* between each pair of consecutive Bragg planes measured along the *X*-axis (Figure A1) is given by [49]:

(A1) $$d_{x}=\frac{\lambda_{r}}{\sin\theta_{r}\;-\sin\theta_{o}}$$

where *θ_o_* and *θ_r_* are the angles formed by the object and reference beams with the normal to the hologram, respectively. In this equation, the signs of the angles are positive clockwise and negative counterclockwise, as shown in Figure A1.

The spatial frequency *f* is the number of interference fringes per unit length, measured along the *X*-axis (see Figure A1). In holography, the spatial frequency *f* is usually expressed in lines/mm (fringes/mm) and is calculated as [49]:

(A2) $$f=\frac{1}{d_{x}}$$

The spacing between two Bragg planes measured perpendicular to them is the grating period Λ. From Figure A1 it is easy to verify that the grating period is:

(A3) $$\Lambda =d_{x}\cos\phi$$

where *ϕ* is the angle formed by the Bragg planes with the normal to the hologram (Figure A1) [49].

For symmetric holographic gratings (Figure A2), which are those in which *θ_o_* = −*θ_r_* (the sign convention defined in Figure A1), the inclination angle of the Bragg planes is *ϕ* = 0° [37] and from Equation (A3) we get Λ = *d*_x_. Considering Equation (A1) and that for symmetric gratings it has that sin *θ_o_* = −sin *θ_r_*, we obtain:

(A4) $$2\Lambda \sin\theta_{r}=\lambda_{r}$$

which is Bragg’s law [48] for symmetric holographic transmission gratings. From Equation (A2), the spatial frequency is:

(A5) $$f=\frac{1}{\Lambda }=\frac{2\sin\theta_{r}}{\lambda_{r}}$$

In volume phase holograms stored in photopolymers, the light interference pattern is stored by modulating the refractive index of the material. For the symmetric grating shown in Figure A2 the refractive index *n*(*x*) varies periodically along the *X*-axis as follows [48]:

(A6) $$n\left(x\right)=n+\Delta n\cos\left(\frac{2nx}{\Lambda }\right)$$

where *n* is the average refractive index of the material, Λ is the grating period and Δ*n* is the index modulation.

Finally, to determine whether a holographic grating is or not a volume hologram, the dimensionless *Q*-factor is used, whose value is [48]:

(A7) $$Q=\frac{2\pi \lambda_{r}d}{n\Lambda^{2}}$$

All the parameters appearing in it have already been previously defined. When a hologram has *Q* > 10, it means that it is a volume hologram [49].
